# The association of serum vitamin K2 levels with Parkinson's disease: from basic case-control study to big data mining analysis

**DOI:** 10.18632/aging.103691

**Published:** 2020-08-29

**Authors:** Yan-Xia Yu, Xiao-Dan Yu, Qin-zhang Cheng, Lian Tang, Ming-Qiang Shen

**Affiliations:** 1 Department of Pharmacy, The Affiliated Suzhou Hospital of Nanjing Medical University, Suzhou Municipal Hospital, Suzhou 215002, China; 2 Department of Neurology, The Affiliated Suzhou Hospital of Nanjing Medical University, Suzhou Municipal Hospital, Suzhou 215002, China

**Keywords:** vitamin K2, Parkinson's disease, neuroinflammation, neurodegenerative disease, coagulation cascades signal

## Abstract

Although it is known that inflammation is involved in Parkinson’s disease (PD) pathogenesis and vitamin K2 (VK2) has anti-inflammatory effects, to date few studies have been reported on the relationship between VK2 and PD development. Herein we presented a case-control study involving 93 PD patients and 95 healthy controls. Overall, the serum VK2 level of PD patients (3.49 ± 1.68 ng/ml) was significantly lower than that of healthy controls (5.77 ± 2.71 ng/ml). When the PD patients were stratified by disease progression, we observed that the serum VK2 level of late stage patients was further decreased to 3.15 ± 1.18 ng/ml while the serum VK2 level of early stage patients was 3.92 ± 2.09 ng/ml. Furthermore, the curve analysis showed that the serum VK2 level decreased gradually with the increment of PD Hoehn-Yahr (H-Y) stage. We also confirmed the dysregulated inflammatory responses and coagulation cascades in PD patients by public dataset, which are associated to the decreased VK2 level. In summary, we found the serum VK2 level in PD patients is lower than that in healthy controls. The decrease of VK2 level may be related to the occurrence and progression of PD by loosening the regulation of inflammatory responses and coagulation cascades signal.

## INTRODUCTION

PD is one of the most common neurodegenerative diseases, but its pathogenesis has not been well-established. Given previous studies, the pathogenesis of PD is mainly related to oxidative stress, mitochondrial dysfunction, abnormal protein aggregation and inflammation. Among these dysregulations, neuroinflammation plays one of the most important roles in PD progression [1]. It has been well-known that there were a great amount of reactive HLA-DR and CD11b-positive microglia in substantia nigra of PD patients, suggesting that inflammatory reaction might be involved in PD [2]. In addition, recent studies have shown the up-regulation of genes encoding inflammatory factors in the brain of patients with PD [3]. Autopsy results also revealed a large number of activated microglia and elevated cytokines in the substantia nigra compact area of PD patients [4]. Furthermore, chronic inflammation in the brain of PD patients has been suggested to be the cause of other pathological manifestations. A previous study found that the incidence of PD was significantly reduced in people who took non-steroidal anti-inflammatory drugs for a long time [5]. Experiments conducted in animal models have also proved that inflammation contributes to the pathogenesis of PD [6–8].

In the past few years, various pathways have come to light that could link dopamine-dependent oxidative stress and microglial activation, finally ascribing a pathogenic trigger to the chronic inflammatory response characteristic of PD [9]. Studies have shown that some vitamins, such as vitamin C and vitamin E, have antioxidant or anti-inflammatory effects, and have some therapeutic or improving effects on neurodegenerative diseases, such as AD and PD [10]. The study also confirmed that vitamin K deficiency plays an important role in the pathogenesis of AD, and vitamin K supplementation may play a role in the prevention and treatment of AD. However, there are few reports on the correlation between vitamin K and PD.

Natural vitamin K includes Vitamin K1 and K2. VK2 is a member of family of isoprenol-like residual naphthoquinones, which has broad applications, such as treating osteoporosis, anti-cancer and inhibiting vascular calcification [11]. Epidemiological investigation showed that vitamin K concentration in human plasma was negatively correlated with various biomarkers of inflammatory factors [12]. *In vitro* and animal experiments further support that vitamin K can suppress the production of pro-inflammatory cytokines [11, 13, 14]. What’s more, previous studies have confirmed that VK2 can inhibit the formation of rotenone-activated microglia and inflammatory factors [15]. These results prove the anti-inflammatory effects of vitamin K and its potential in treating inflammatory-related diseases.

VK2 is known to be widely distributed in human tissues. After oral administration of VK2 to rats, VK2 could be distributed in liver, kidney, spleen and brain within 24 hours [16], making it an ideal candidate in terms of drug delivery. Given that VK2 can pass through the blood-brain barrier and inhibit inflammatory response, we speculate that it may inhibit brain neuroinflammation and play a preventive and therapeutic role in neurodegenerative diseases such as PD. Although it has been known that inflammation is involved in PD pathogenesis and VK2 has anti-inflammatory effects, there are few studies on the relationship between VK2 and PD development in Parkinson's population. Our previous study found that VK2 can significantly improve the behavioral disorder of PD rats induced by rotenone *in vitro* (unpublished). VK2 can inhibit rotenone-induced activation of microglial BV2 cells by repressing the expression of inflammatory factors and nuclear transportation of NF-κ B. VK2 can also restore mitochondrial membrane potential and protect SH-SY5Y neurons by inhibiting the secretion of inflammatory mediators of microglia [15]. Therefore, this study will explore whether the VK2 level of PD patients is related to the occurrence and progression of PD.

## RESULTS

### Baseline characteristics

The PD patients and controls did not differ with respect to age and gender distribution. A total of 93 cases in the PD group were included in this study, with the age of 69.18 ± 10.429 years old and the gender ratio (male/female) of 42/51. Ninety-five healthy controls were recruited with the age of 66.42 ± 9.971 years old and the gender ratio (male/female) of 52/43. The smoke and alcohol conditions were also listed and showed no significant difference between PD patients and healthy controls (Table 1).

**Table 1:** Characteristics of PD patients and controls.

	**Controls(n=95)**	**PD patients(n=93)**	**t/F**	**P**
Age (years)	66.42 ± 9.971	69.18 ± 10.429	1.856	0.065
Gender (males/females)	52/43	42/51	1.724	0.189
Smoke (cigarette/year)	247.11±556.63	99.89 ± 250.87	0.577	0.447
Alcohol (g/year)	128.95±333.54	27.15 ± 85.70	0.610	0.435

### The serum levels of VK2 in PD group and control group

Our results showed that the serum VK2 levels in PD patients were significantly lower than those in controls. The serum levels of VK2 were 3.49 ± 1.68 ng/ml in PD group and 5.77 ± 2.71 ng/ml in control group (t = -6.930, P < 0.0001).

After grouping by gender, the mean value of VK2 level in the PD male group was 3.35 ±1.58ng/ml and that in the control male group was 5.50 ± 2.67 ng/ml. There was a significant difference between these two groups based on student’s t test. Similarly, VK2 level in the PD female group (3.61 ± 1.76 ng/ml) was also significantly lower than the female health control group (6.10 ± 2.75 ng/ml). In contrast, there was no significant difference of VK2 level between men and women in PD group or control group (P > 0.05).

PD patients and healthy controls were also stratified into two groups based on age. For both non-elderly group (< 65 years old) and elderly group (≥65 years old), we observed significantly lower VK2 level in PD patents. As for the patients or healthy controls between non-elderly group and elderly group, there is no significant difference of serum VK2 level (Table 2).

**Table 2:** VK2 serum levels in PD group and control group.

	**Control group(n=95)**	**PD group(n=93)**	**t/F**	**P**
VK2 level (ng/ml)	5.77±2.71	3.49 ± 1.67	-6.930	0.000
Males	5.50±2.67	3.35±1.58	-4.859	0.000
Females	6.10±2.75	3.61±1.768	-5.125	0.000
<65 years old	6.34±2.69(n=44)	3.93±1.96 (n=22)	-3.732	0.000
≥65years old	5.28±2.65(n=51)	3.35±1.57(n=71)	-5.026	0.000

### The serum levels of VK2 in different stage of PD group

**Figure 1:**
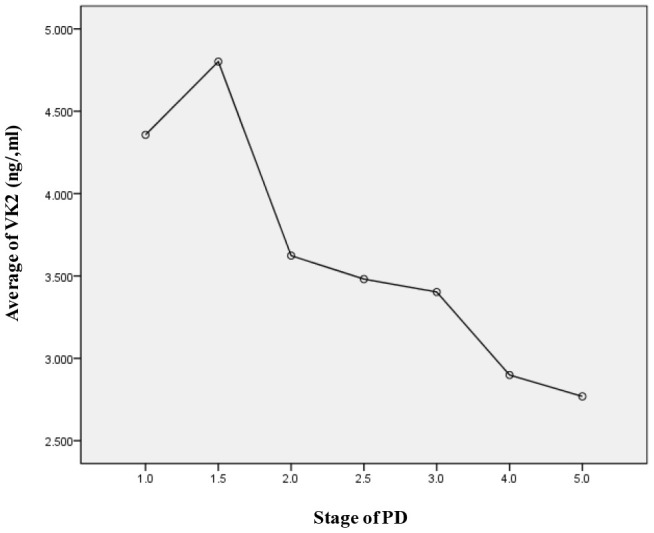
**The serum levels of VK2 in different H-Y stages of PD. Serum VK2 level is negative corelated with the PD stage.**

PD patients were further stratified into early stage group and middle-late stage group based on H-Y stages. We found that the serum level of VK2 in the early stage group (3.92 ± 2.09 ng/ml) was significantly higher than that in the middle-late stage group (3.15 ± 1.18 ng/ml). And this observation was irrelevant to the age of patients (Table 3). We further did curve analysis and observed that the VK2 level was anti-correlated to the H-Y stage (Figure 1). Altogether, our research showed that with the progression of PD, the serum VK2 level was gradually decreased.

**Table 3:** The serum levels of VK2 in different courses of PD group.

	**early stage of PD (n=41)**	**middle-late stage of PD group (n=52)**	***t***	***P***
VK2 level (ng/ml)	3.92±2.09	3.15±1.18	-2.102	0.040
<65 years old	4.23±2.21 (n=16)	3.14±0.65 (n=6)	1.780	0.091
≥65 years old	3.72±2.03 (n=25)	3.15±1.23 (n=46)	1.466	0.147

### Elevated inflammatory response in PD patients

**Figure 2:**
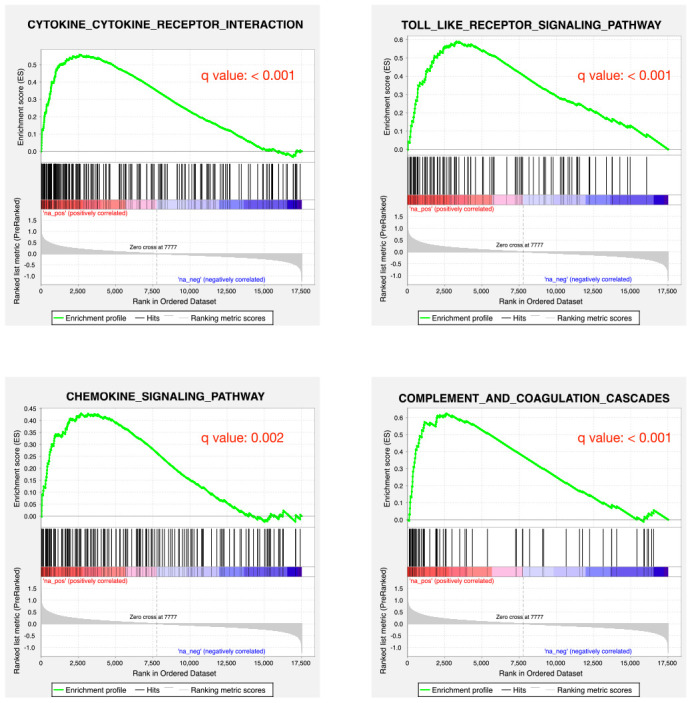
**Inflammatory responses are up-regulated in PD patients.** GSEA plots showing the up-regulation of cytokine-cytokine receptor interaction, toll-like receptor signaling pathway, chemokine signaling pathway and complement and coagulation cascades pathways.

**Figure 3:**
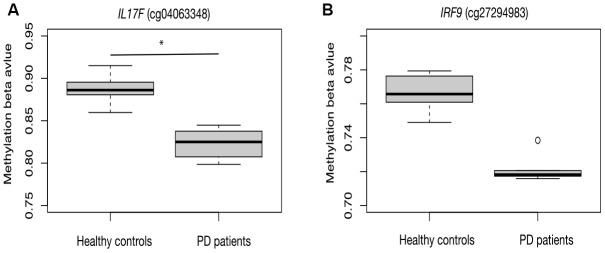
**Hypomethylation of inflammation related genes in PD patients.** CpG sites in the promoter region of IL17F (**A**) and IRF9 (**B**) are hypomethylated in PD patients.

As a proof-of-principle, we next examine whether the inflammatory reaction is indeed dysregulated in PD patients. RNA-seq data from the PD patients’ post-mortem BA9 brain tissue for Parkinson's disease and neurologically normal individuals were collected from public dataset (GSE68719) and analyzed with STAR and DEseq2. We observed 231 up-regulated and 220 down-regulated genes in PD patients. We further did GSEA analysis and found that many pathways related to inflammation response were enriched in up-regulated genes, such as cytokine-cytokine receptor interaction, toll-like receptor signaling pathway, chemokine signaling pathway and complement and coagulation cascades (Figure 2). This result confirmed that there were elevated inflammatory reactions in PD patients. Given previous studies suggesting DNA methylation is involved in PD development, we also investigated whether DNA methylation change was associated with the elevated inflammatory response observed in PD patients. We collected public 450K methylation array data of brain grey matter from PD patients and healthy controls. As expected, we found hypomethylated CpG sites annotated to the genes related to immune and inflammatory responses, such as IRF9, and IL17F (Supplementary Table 1, Figure 3). Given these results, we speculate that VK2 may play a role in preventing PD by repressing neuroinflammation. And the inhibited expression of inflammation factors could be achieved through epigenetic regulation, such as DNA methylation. However, more evidences are required before a solid conclusion can be draw.

### Elevated coagulation cascades signal in PD patients

**Figure 4:**
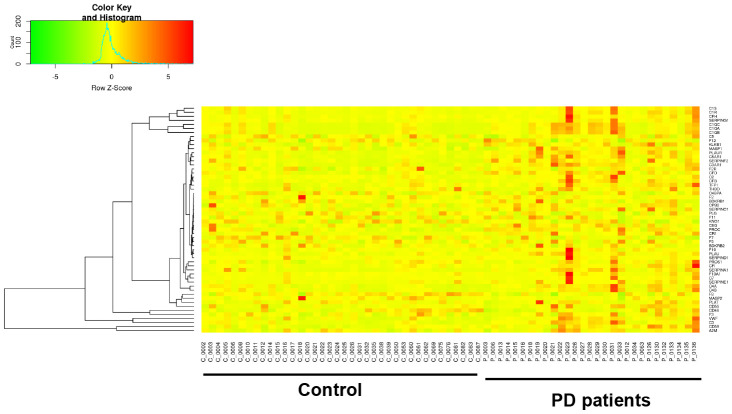
**Gene expression heatmap of coagulation cascade gene clusters in PD patients and controls.**

**Figure 5:**
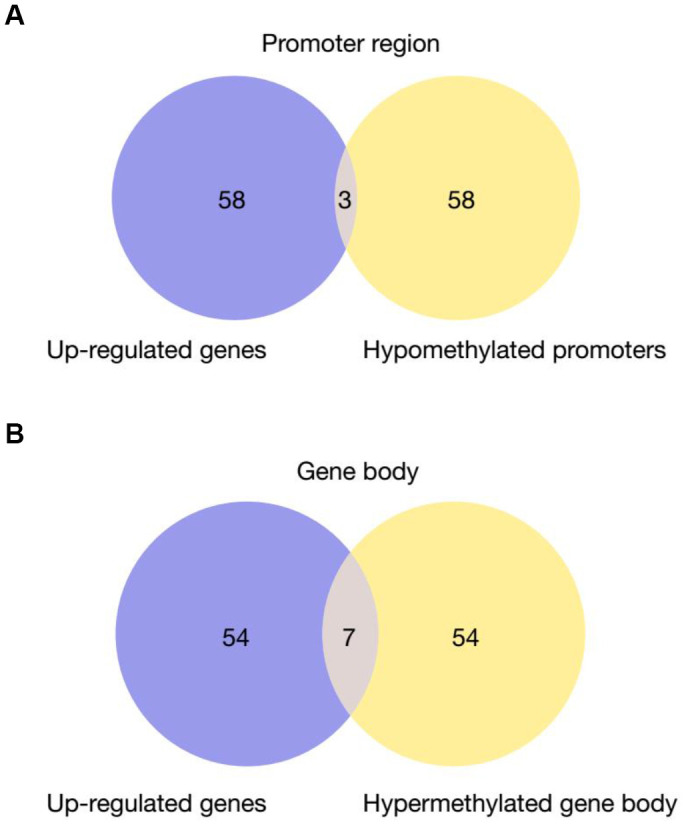
**Venn Diagram of up-regulated genes in PD patients that have hypomethylated promoters** (**A**) or gene bodies (**B**) Fisher’s exact test is used for the comparison of preference of methylation at gene body versus promoter. P-value = 0.0029.

**Figure 6:**
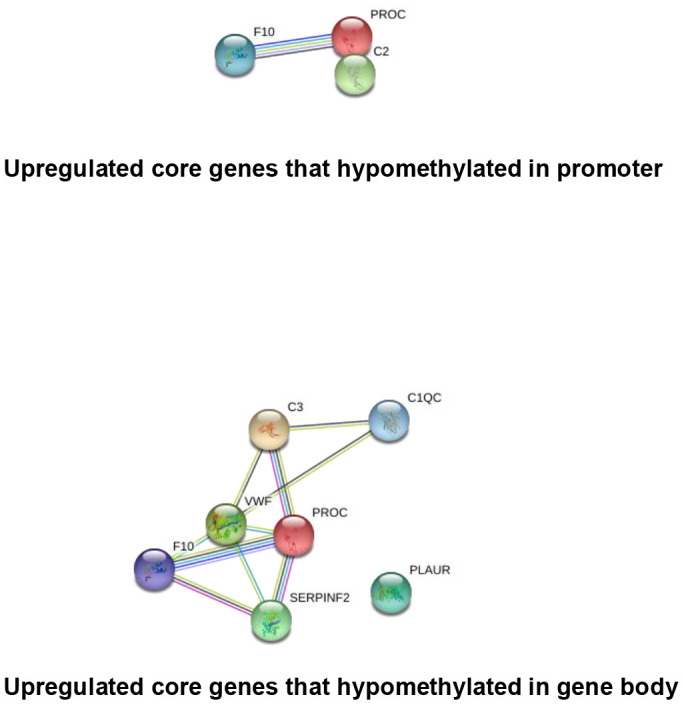
**String network analysis of the core genes that directly regulated by DNA methylation.**

It has been widely established that VK2 plays crucial role in blood clotting and coagulation. In our collected dataset, we did find an enrichment of upregulated coagulation genes only in PD patients (Figures 2, 4). This result indicates that VK2 may contribute to the process of PD through regulating coagulation cascades. We next examined the regulation of these ‘core’ coagulation cascade genes (Enriched in GSEA) in terms of DNA methylation *via* using our collected DNA 450K methylation array data. Interestingly, in the PD patients, we identified 3 upregulated coagulation genes have hypomethylated promoters in the PD patients, whereas 7 upregulated coagulation genes are hypomethylated in the gene body regions (Figure 5A, 5B). These results suggested that the upregulation of these 10 coagulation genes may be directly regulated *via* DNA methylation. Moreover, these results also implied that the methyl-regulation of coagulation gene clusters in PD patients may have preference on gene bodies (Fisher’s exact test, p = 0.0029). Finally, we did the network analysis on these methyl-regulated coagulations genes in order to identify their connections. Interestingly, we demonstrated that these gene-body-regulated coagulation gens were in the same network (Figure 6). In conclusion, we found the VK2 may be associated with PD process through regulating coagulation cascades.

## DISCUSSION

VK2 has been found to play a neuroprotective role in nervous system diseases, but there are few studies on the relationship between VK2 and PD. Recent studies have found that natural vitamin K can improve mental activity and cognition by participating in the synthesis of sphingolipids to nourish brain cells [17]. The elderly people who take vitamin k every day are shown to have better cognition and behavior [18]. Hadipour E et al [19] found that VK2 has anti-apoptosis and anti-oxidation effects, and suggested that VK2 may be a valuable protective candidate to prevent the progression of Alzheimer's disease by inactivating p38 MAP kinase pathway. In the PD model of Drosophila, when the mutation of UBIAD1/Heixgene causes serious damage to mitochondria, VK2 can protect mitochondria by producing more effective ATP through electron transportation. Thus VK2 may constitute a promising compound to treat mitochondrial pathology, also in PD patients suffering from Pink1 or Parkin deficiency [20].

Preliminary *in vitro* and *in vivo* experiments lead us to speculate that VK2 may have a therapeutic effect on Parkinson's disease [15]. In order to further study the relationship between VK2 and PD, we compared the serum VK2 level of PD patients with that of the control group. We observed significantly lower serum VK2 level in PD patient group, consistent with our previous assumption that VK2 level is related to PD pathogenesis. This result indicates that VK2 may related to the occurrence and development of PD and can be potential biomarker for PD diagnosis or prognosis.

Since the occurrence and development of PD are related to age factors, we divided age into non-elderly group (< 65 years old) and elderly group (≥ 65 years old) based on current conventions. We found that there was no significant difference in VK2 level between the non-elderly group and the elderly group of PD patients. In contrast, we observed significant differences between the two groups of PD patients and the control group. This result confirmed that the content of VK2 had no significant relationship with age, but with PD itself.

We also examined whether the level of VK2 was related to gender. The results showed that there was no significant difference of serum VK2 level between different genders in PD patients or healthy controls, suggesting that gender differences had no effect on the level of VK2. And for either male or female patients, their serum VK2 levels were significantly higher than the healthy controls. Collectively, these results suggested that the decreased serum level of VK2 in PD patients has no relevance to gender.

We then studied the relationship between VK2 level and the progression of PD. Since PD is a chronic progressive disease, the clinical severity of which is evaluated by H-Y stage table, we then stratified PD patients into early stage and middle-late stage group. H-Y1-2.5 level is defined as early stage, H-Y 3-5 level is defined as middle-late stage. Interestingly, we found that the level of VK2 in the serum of patients with middle-late stage PD was significantly lower than that of patients with early stage PD, indicating that the level of VK2 in patients is decreasing with the development of PD.

Finally, we confirmed the elevated inflammatory responses in PD patients by public RNA-seq dataset. Based on the GSEA analysis, several immune and inflammation related pathways were highly enriched in the up-regulated genes in PD patients. Several inflammation related genes were also hypomethylated, suggesting that epigenetic regulation may play an important role in regulating inflammation of PD patients. Moreover, we also identified the potential association between VK2 and coagulation signal. We hypothesized that the long-term low level of VK2 may be a reason that could increase the coagulation signal, thus contributing to the PD process. However, more evidences and careful examinations are required before proving the causal relationship between VK2 and PD development.

## CONCLUSIONS

In summary, our findings shed light on the unknown relationship between serum VK2 levels and PD pathogenesis and progression. We found that the VK2 level in PD patients was significantly lower than that in healthy controls. The decreased VK2 level may be related to the occurrence and development of PD. This is the first time that a pronounced lower serum VK2 level in the PD patients is demonstrated. Since inflammatory response is one of the important pathogenesis of PD and VK2 has anti-inflammatory effects, we speculate that PD patients may be caused by the lack of VK2 *in vivo*, which leads to the occurrence and aggravation of inflammatory response, dysregulation of coagulation cascades, and eventually the occurrence of PD. The associations between VK2 and PD found in this study should be further investigated in the future with well-designed animal experiments and large-scale clinical studies.

### Limitations

There were certain limitations in this study. Due to the short duration of the study, the observation method did not use prospective study to dynamically observe the serum VK2 concentration of each PD patient with the course of disease. The number of research cases is relatively small, and the baseline data collected by patients is not complete. Therefore, this study is simply presented as a proof-of-principle to show the correlation between the VK2 level and PD. However, more baseline data should be collected, and multicenter prospective studies will be conducted in the future. If similar results can be observed, the intervention study of VK2 on PD patients will also be carried out.

## MATERIAL AND METHODS

### Study sample

During March 2016 to March 2019, ninety-three patients with PD were consecutively recruited from Suzhou municipal hospital in Jiangsu, China. All the diagnoses were in accordance with the British Brain Bank Diagnostic Criteria [21]. Exclusion criteria: (1) secondary PD syndrome caused by poisoning, brain trauma, drugs, cerebrovascular disease, encephalitis and other causes; (2) multiple system atrophy, progressive supranuclear palsy, corticobasal ganglia degeneration and other PD superposition syndrome; (3) associated with tumors, liver and kidney dysfunction, inflammation and other diseases. The healthy controls were selected from the same period in the physical examination center of the same hospital, without tumors, inflammation, liver and kidney dysfunction and other diseases.

### Data collection

Name, sex, age smoke and alcohol condition, course of disease and medication of PD patients and control group were recorded, and the severity of motor symptoms was assessed by H-Y stage table. The course of PD can be divided into early and middle-late stages. H-Y1-2.5 is defined as early stage, and H-Y3-5 is defined as middle-late stage [15, 22, 23].

### Detection of blood samples

Venous blood samples (≤5 mL) for determination of serum VK2 levels were collected from all participants. VK2 in plasma was detected by enzyme-linked immunosorbent assay (LBC Reagent purchased by Shanghai Jianglai Biotechnology Co. Ltd., detection instrument: TECAN Free Evalyzer automatic enzyme analyzer; made in Switzerland). Detection unit: ng/ml.

### RNA-seq data analysis

Raw RNA-seq sequencing data was downloaded from public dataset (Gene Expression Omnibus). GEO accession number is GSE68719. After removing adapter and low-quality sequence by cutadapt (version 2.7), the sequencing reads were mapped to hg19 human reference by STAR (version 2.5.1). Read counts for each gene were quantified by HTSeq-count (version 0.11.1). Finally, DEseq2 was used for identifying the differential expressed genes.

### DNA methylation analysis

Processed 450K DNA methylation array data was downloaded from public dataset (GSE57360). ‘ChAMP’ R package was used to normalize the downloaded beta value and identify the differential methylated CpG sites (DMC). The DMCs were then annotated to the nearest genes. String network analysis is used for core gene network analysis (https://string-db.org/).

### Statistical analysis

All patients were divided into two groups based on age with the cut off at 65 years old (the <65 years old subgroup and ≥ 65 years old subgroup) and all PD patients were divided into the early stage subgroup and the middle-late stage subgroup according to their courses. All continuous data were presented with mean values, standard deviations (SDs).

The Kolmogorov–Smirnov test was used to assess the normality of distribution of the continuous data. The independent sample student t-test and the Kruskal–Wallis were used to compare continuous data with and without normal distribution, respectively, between the PD group and the control group or between other subgroups. The Pearson χ^2^ test or Fisher’s exact test were used to compare gender between the PD group and the control group. Data analyses were performed using SPSS 25.0 (SPSS, IBM Corp., Armonk, NY, USA). A *P* value < 0.05 was considered to indicate statistical significance.

### Ethical considerations

Written informed consent was obtained from allparticipants. This study was approved by the Ethics Committee of the Affiliated Suzhou Hospital of Nanjing Medical University, Suzhou Municipal Hospital (number K2017007).

## Supplementary Material

Supplementary Table 1
